# Intensive vs Conventional Blood Pressure Control After Thrombectomy in Acute Ischemic Stroke: A Systematic Review and Meta-Analysis

**DOI:** 10.1001/jamanetworkopen.2024.0179

**Published:** 2024-02-22

**Authors:** Sherief Ghozy, Ali Mortezaei, Mohamed Elfil, Mariam Abdelghaffar, Hassan Kobeissi, Mohammad Aladawi, Alzhraa S. Abbas, Sandeep S. Nayak, Ramanathan Kadirvel, Alejandro A. Rabinstein, David F. Kallmes

**Affiliations:** 1 Department of Neurosurgery, Mayo Clinic, Rochester, Minnesota; 2 Department of Radiology, Mayo Clinic, Rochester, Minnesota; 3 Gonabad University of Medical Sciences, Gonabad, Iran; 4 Department of Neurological Sciences, University of Nebraska Medical Center, Omaha; 5 Evidence-Based Practice Center, Kern Center for the Science of Healthcare Delivery, Mayo Clinic, Rochester, Minnesota; 6 Department of Internal Medicine, Yale New Haven Health Bridgeport Hospital, Bridgeport, Connecticut; 7 Department of Neurology, Mayo Clinic, Rochester, Minnesota

## Abstract

**Importance:**

Endovascular thrombectomy (EVT) is standard treatment for acute ischemic stroke (AIS) due to large-vessel occlusion (LVO), but optimal post-EVT blood pressure (BP) control remains debated.

**Objective:**

To assess the association of different systolic BP targets following EVT with functional outcomes, mortality, and complications in patients with AIS due to LVO.

**Data Sources:**

Systematic review and meta-analysis of databases (PubMed, Embase, Web of Science, Scopus, and Cochrane Library) to September 8, 2023.

**Study Selection:**

Inclusion criteria consisted of randomized clinical trials examining post-EVT management of systolic BP in patients with AIS and LVO comparing intensive vs conventional targets. Nonrandomized studies, observational studies, noninterventional trials, meeting abstracts, duplicate studies, studies with overlapping data, and non–English language studies were excluded. Two authors independently applied these criteria through a blinded review, with discrepancies resolved through consensus. The risk of bias in the included studies was assessed using the revised tool for assessing risk of bias in randomized trials.

**Data Extraction and Synthesis:**

This study adhered to the Preferred Reporting Items for Systematic Reviews and Meta-Analyses (PRISMA) reporting guideline. Three authors extracted data regarding study characteristics, baseline patient data, and outcomes of interest. The pooled data were analyzed using a random-effects meta-analysis.

**Main Outcomes and Measures:**

Rates of functional independence, 90-day mortality, symptomatic intracranial hemorrhage, and hypotensive events.

**Results:**

A total of 4 randomized clinical trials with 1571 initially enrolled patients were included in the analysis. Lower functional independence rates were observed in the intensive control group (relative risk [RR], 0.81 [95% CI, 0.67-0.98]). No significant differences were found in 90-day mortality (RR, 1.18 [95% CI, 0.92-1.52]), symptomatic intracranial hemorrhage (RR, 1.12 [95% CI, 0.75-1.67]), or hypotensive events (RR, 1.80 [95% CI, 0.37-8.76]). There was minimal heterogeneity among the studies included in the functional independence outcome (*I*^2^ = 13% and τ^2^ = 0.003), which was absent among other outcomes (*I*^2^ = 0 and τ^2^ = 0).

**Conclusions and Relevance:**

These findings suggest that intensive post-EVT BP reduction does not yield benefits and may carry risks. While awaiting the results of additional ongoing trials, a conservative BP management strategy after endovascular recanalization is favored in daily practice.

## Key Points

**Question:**

What are the implications of intensive vs conventional blood pressure (BP) control following endovascular thrombectomy in patients with acute ischemic stroke due to large-vessel occlusion?

**Findings:**

This systematic review and meta-analysis of 4 randomized clinical trials with 1571 initially enrolled participants revealed lower functional independence rates in intensive compared with conventional BP control. No significant differences were observed in 90-day mortality, symptomatic intracranial hemorrhage, or hypotensive events.

**Meaning:**

These findings suggest that intensive BP reduction post thrombectomy does not offer benefits and may pose risks and that a conservative management strategy after endovascular recanalization should be favored in daily practice until additional ongoing trials are completed.

## Teaser

This systematic review and meta-analysis compares conventional and intensive systolic blood pressure (BP) control after endovascular thrombectomy among patients with acute ischemic stroke due to large-vessel occlusion.

## Introduction

Endovascular thrombectomy (EVT) has become the standard of care for patients with acute ischemic stroke (AIS) due to large-vessel occlusion (LVO). Several randomized clinical trials (RCTs) and meta-analyses^1,2,3^ have demonstrated that EVT significantly improves functional outcomes and reduces disability compared with medical management alone in these patients. However, despite high rates of recanalization with EVT, a substantial proportion of patients still have poor functional outcomes.^4,5^ Optimization of periprocedural management, including blood pressure (BP) control following successful recanalization, may help further improve outcomes in patients with AIS-LVO undergoing EVT.

Contemporary recommendations derived from the systolic BP (SBP) guidelines established for intravenous thrombolysis advocate for maintaining a post-EVT SBP target of less than 180 mm Hg.^6^ However, observational studies have suggested an association between post-EVT elevated BP and risks of intracranial hemorrhage (ICH) and poor functional outcomes, especially in patients with successful recanalization.^7,8,9,10,11^ This has led to a trend toward intensive BP lowering after EVT, with an SBP target less than 140 or even less than 120 mm Hg.^6^ On the other hand, recently published clinical trials^12,13^ indicate that an overly aggressive reduction of SBP after EVT may have a detrimental effect on functional outcomes while affording no significant reductions in rates of ICH following successful recanalization. Therefore, we performed a systematic review and meta-analysis of RCTs comparing different BP targets after EVT for AIS-LVO.

## Methods

### Search Strategy

This study adhered to the Preferred Reporting Items for Systematic Reviews and Meta-Analyses (PRISMA) reporting guideline. To conduct our systematic review, we used AutoLit software, version 1.70.4 (Nested Knowledge). The protocol was registered with Nested Knowledge with the nest identification 7736. We conducted a thorough search across multiple databases, including PubMed, Embase, Web of Science, Scopus, and Cochrane Library, encompassing articles from their inception (ie, the earliest year for each database) to September 8, 2023. These searches were tailored to each database using a diverse array of keywords and medical subject heading terms. In PubMed, the search included terms such as *blood pressure*, *endovascular therapy*, *endovascular thrombectomy*, *stroke*, and *trial*. Similar terms were used in Scopus, Embase, Web of Science, and the Cochrane Library to ensure a comprehensive search across multiple databases. A comprehensive overview of search strategies is found in eTable 1 in Supplement 1.

### Screening Process and Eligibility Criteria

Two authors (M. Abdelghaffar and H.K.) independently conducted the initial screening of the titles and abstracts through a blinded review, adhering to predefined criteria. Subsequently, any studies that passed the initial screening underwent a thorough full-text review. Throughout both screening stages, the senior author (D.F.K.) actively participated in resolving any conflicts in decision-making.

We included all RCTs aligned with our predefined criteria, encapsulated within the framework of PICO (patient or problem; intervention or exposure; comparison or control; and outcome). The population of interest consisted of patients with AIS treated with mechanical thrombectomy. The intervention examined was intensive SBP control, with the comparator as conventional SBP control. The outcomes of interest encompassed a modified Rankin Scale (mRS) score of 0 to 2 at 90 days, mortality at 90 days, symptomatic ICH (sICH), and hypotensive events. Nonrandomized studies, observational studies, noninterventional trials, meeting abstracts, duplicate studies, studies with overlapping data, and non–English language studies were excluded.

### Data Extraction

To ensure meticulous precision, 3 authors (A.M., M.E., and A.S.A.) used the AutoLit software, version 1.70.4 for the purpose of data extraction. The extracted information encompassed essential elements such as study characteristics, baseline patient data, and the aforementioned outcomes of interest. Following the initial data extraction process, a third author (S.G.) conducted a thorough review of the extracted data to ensure accuracy and resolve any discrepancies, ensuring a consensus was reached among the team.

### Risk of Bias Assessment

Two reviewers (A.M. and M. Aladawi) conducted a comprehensive evaluation of bias risk using the revised tool for assessing risk of bias in randomized trials (RoB 2).^14^ This assessment tool is designed to assess bias risk in specific domains, encompassing the following: bias arising from the randomization process (D1), bias due to deviations from intended interventions (D2), bias due to missing outcome data (D3), bias in measurement of the outcome (D4), and bias in selection of the reported result (D5). The RoB 2 tool provides an overall judgment of bias risk, which includes the categories low risk of bias, high risk of bias, and some concerns.^14^

### Statistical Analysis

In our study, we performed random-effects meta-analysis using R software, version 4.3.1 (R Project for Statistical Computing) and the meta statistical package.^15^ We calculated the risk ratios (RRs) for binary variables and the mean differences for continuous variables, each accompanied by their respective 95% CIs. To calculate the 95% CI of the random-effects estimate, we used a restricted maximum likelihood estimator with the modification of the Hartung-Knapp-Sidik-Jonkman variance correction^16^ by Jackson et al^17^ and the hybrid method 2 in Jackson et al as an ad hoc correction.^17,18,19^ Furthermore, we assessed the presence of heterogeneity in the data using Cochran Q and *I*^2^ tests, considering a significance level of 2-sided *P* < .05 for the Q statistic and *I*^2^ values above 50% as indicative of significant heterogeneity. To investigate the influence of each study on the overall effect-size estimate, we conducted influence analysis using the leave-one-out method. Due to the limited number of studies included in each analysis (<10), we were unable to conduct an Egger regression test to assess publication bias or perform meta-aggression.^20^

## Results

### Study Selection and Evaluation

**Figure 1.**
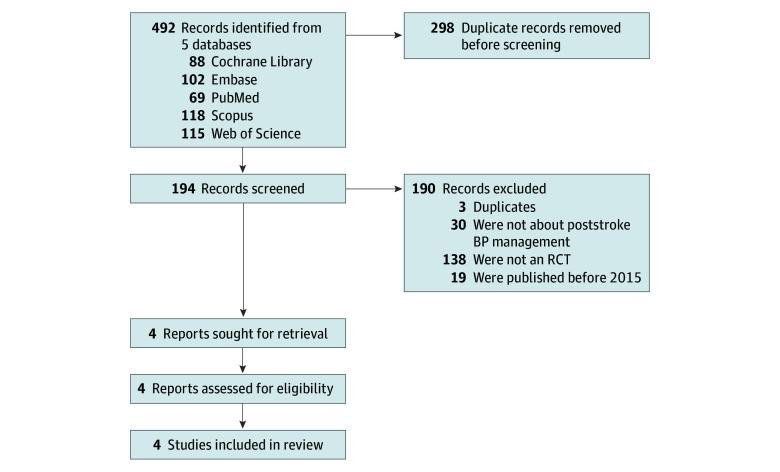
Study Flow Diagram BP indicates blood pressure; RCT, randomized clinical trial.

The initial database search yielded a total of 492 records, including 88 from the Cochrane Library, 102 from Embase, 69 from PubMed, 118 from Scopus, and 115 from Web of Science. After conducting a thorough screening of titles and abstracts and the removal of duplicate entries, we refined the list to encompass 194 studies. During the title and abstract screening phase, 190 records were excluded, leaving 4 records that underwent a full-text article assessment to ascertain eligibility. None of the full-text articles failed to meet the inclusion criteria, resulting in the inclusion of all 4 studies in our quantitative synthesis.^12,13,21,22^ The studies were published between 2021 and 2023 and were conducted in China, France, South Korea, and the US. A comprehensive depiction of the selection process can be found in Figure 1. A total of 1571 initially enrolled patients were included in this analysis.

### Study Characteristics

The fundamental attributes of the included studies are comprehensively outlined in eTable 2 in Supplement 1. A detailed examination of various aspects of each included study was conducted to assess any variations in baseline characteristics. These aspects encompassed study design, patient count, age distribution, sex representation, medical history, race and ethnicity, antihypertensive drugs used upon admission, occlusion site, etiology of LVO, use of general anesthesia, number of intravenous thrombolysis procedures performed, baseline National Institutes of Health Stroke Scale score, Alberta Stroke Program Early CT (Computed Tomography) score, adverse events, hemorrhagic transformation, and infarction volumes. Moreover, a comprehensive presentation of BP-related details and measurements can be found outlined in eTable 3 in Supplement 1.

### Risk of Bias

A compilation of the 4 studies included in our analysis, along with their respective evaluations of bias across various domains using the RoB 2, can be accessed in eFigures 1 and 2 in Supplement 1. Among these studies, our assessment revealed that 3 of them^12,13,21^ had a moderate risk of bias, while one^22^ was categorized as having a low risk of bias. Notably, the domains demonstrating more concerns were bias arising from the randomization process and bias in measurement of the outcome.

### Comparison of Baseline Characteristics

Analyzing the data from the 4 RCTs included in our study, we observed a balance in baseline demographics across all studies, with no significant differences noted in any of the included characteristics. The dichotomous variables examined encompassed male sex, hypertension, diabetes, smoking, hypercholesterolemia, previous stroke or transient attack, atrial fibrillation, coronary artery obstructive disease, antiplatelet and anticoagulant use, intravenous thrombolysis, and the administration of general anesthesia. In addition, we assessed continuous variables such as age, National Institutes of Health Stroke Scale score, infarction volumes, and onset to reperfusion time. A detailed analysis of each variable assessed can be found in eFigures 3 and 4 in Supplement 1.

### Functional Independence 

**Figure 2.**
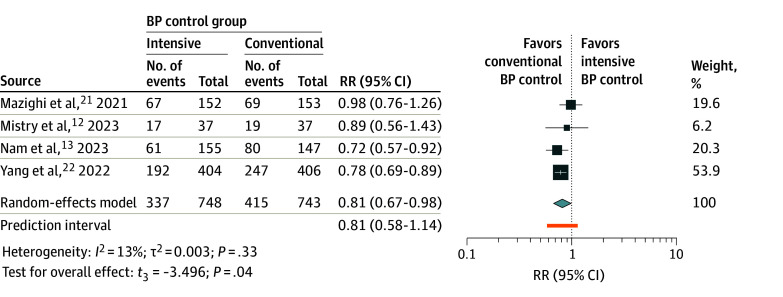
Meta-Analysis Forest Plot for Functional Independence Rates at 90 Days For each individual study, the estimated risk ratio (RR; represented by a square) and the corresponding 95% CI are shown for the rate of functional independence (modified Rankin Scale score range, 0-2) at 90 days in patients with acute ischemic stroke undergoing different blood pressure (BP) management strategies after endovascular thrombectomy. The size of each square is proportional to the study’s weight in the meta-analysis, with larger squares indicating studies with greater influence. The diamond represents the overall pooled estimate; its width denotes the 95% CI around the combined effect size. The *t*_3_ indicates Hartung-Knapp-Sidik-Jonkman variance correction.

Rates of functional independence (mRS scores 0-2) between intensive and conventional BP control among 1491 patients were compared across all 4 studies included.^12,13,21,22^ The rate of 90-day mRS scores of 0 to 2 in the intensive BP control group (337 of 748 [45.1%]) was observed to be lower compared with the conventional BP control group (415 of 743 [55.9%]), resulting in a calculated RR of 0.81 (95% CI, 0.67-0.98; *P* = .04) (Figure 2), indicating a statistically significant difference between both groups. Notably, there was minimal heterogeneity among the studies included, with *I*^2^ = 13% and τ^2^ =0.003.

### 90-Day Mortality

**Figure 3.**
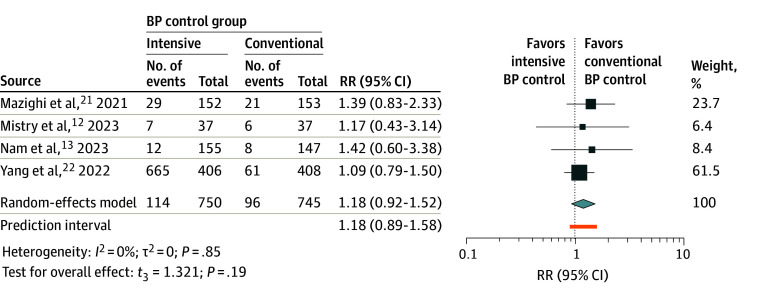
Meta-Analysis Forest Plot for 90-Day Mortality Rates For each individual study, the estimated risk ratio (RR; represented by a square) and the corresponding 95% CI are shown for the 90-day mortality rate in patients with acute ischemic stroke undergoing different blood pressure (BP) management strategies after endovascular thrombectomy. The size of each square is proportional to the study’s weight in the meta-analysis, with larger squares indicating studies with greater influence. The diamond represents the overall pooled estimate; its width denotes the 95% CI around the combined effect size. The *t*_3_ indicates Hartung-Knapp-Sidik-Jonkman variance correction.

The conducted analysis comparing the 90-day mortality rates between patients receiving intensive and conventional BP control included 1495 patients from all 4 studies.^12,13,21,22^ In the intensive BP control group, the 90-day mortality was found to be higher (114 of 750 [15.2%]) than in the conventional BP control group (96 of 745 [12.9%]). However, the calculated RR was 1.18 (95% CI, 0.92-1.52; *P* = .19) (Figure 3), indicating no significant difference between the 2 groups. Importantly, there was no observed heterogeneity among the studies included, as evidenced by *I*^2^ = 0 and τ^2^ = 0.

### Hypotensive Events

**Figure 4.**
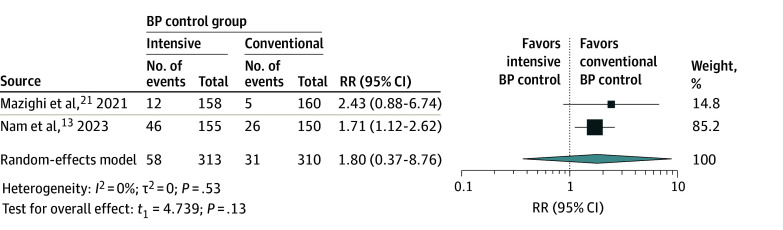
Meta-Analysis Forest Plot for Hypotensive Events For each individual study, the estimated risk ratio (RR; represented by a square) and the corresponding 95% CI are shown for the occurrence of hypotensive events in patients with acute ischemic stroke undergoing different blood pressure (BP) management strategies after endovascular thrombectomy. The size of each square is proportional to the study’s weight in the meta-analysis, with larger squares indicating studies with greater influence. The diamond represents the overall pooled estimate; its width denotes the 95% CI around the combined effect size. The *t*_3_ indicates Hartung-Knapp-Sidik-Jonkman variance correction.

Results from 2 studies^13,21^ involving 623 patients indicated that the group with intensive BP control (58 of 313 [18.5%]) had a higher occurrence of hypotensive events compared with the conventional BP control group (31 of 310 [10.0%]). However, the calculated RR of 1.80 (95% CI, 0.37-8.76; *P* = .13) (Figure 4) did not reach statistical significance. There was no observed heterogeneity among the included studies, as indicated by *I*^2^ = 0 and τ^2^ = 0.

### Symptomatic ICH

**Figure 5.**
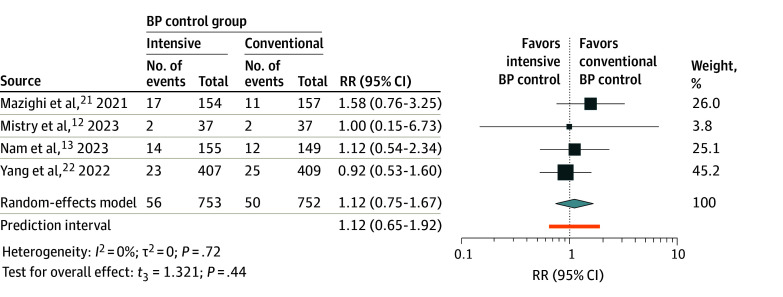
Meta-Analysis Forest Plot for Symptomatic Intracranial Hemorrhage For each individual study, the estimated risk ratio (RR; represented by a square) and the corresponding 95% CIs are shown for the rate of symptomatic intracranial hemorrhage in patients with acute ischemic stroke undergoing different blood pressure (BP) management strategies after endovascular thrombectomy. The size of each square is proportional to the study’s weight in the meta-analysis, with larger squares indicating studies with greater influence. The *t*_3_ indicates Hartung-Knapp-Sidik-Jonkman variance correction. The diamond represents the overall pooled estimate; its width denotes the 95% CI around the combined effect size.

Data on the sICH rate was provided by all 4 studies,^12,13,21,22^ encompassing a total of 1503 patients. In the intensive BP control group (56 of 753 [7.4%]), the sICH rate was found to be higher compared with the conventional BP control group (50 of 752 [6.6%]). The calculated RR of 1.12 (95% CI, 0.75-1.67; *P* = .44) (Figure 5) did not demonstrate statistical significance. No heterogeneity was found among the included studies, as evidenced by *I*^2^ = 0 and τ^2^ = 0.

### Leave-One-Out Influence Analysis

For the functional independence outcome, removing the study by Mazighi et al^21^ resulted in eliminating heterogeneity (*I*^2^ = 0; τ^2^ = 0) and bringing the results to be more significant (RR, 0.77 [95% CI, 0.67-0.89]; *P* = .02); nevertheless, excluding any of the other studies resulted in eliminating significance and increasing heterogeneity (eFigure 5 in Supplement 1). For the 90-day mortality and sICH outcomes, removing any study did not have a tangible effect on significance or heterogeneity (eFigure 5 in Supplement 1). The analysis was not possible for the hypotensive events outcome since it was reported in only 2 studies.

## Discussion

Our systemic review and meta-analysis included 4 RCTs investigating different levels of SBP control after successful reperfusion in patients with AIS-LVO undergoing EVT. The results showed higher functional independence rates in the conventional BP management group compared with the intensive BP management group. However, there were no between-group differences with regard to 90-day mortality, hypotensive events, and sICH. Trends favored the conventional strategy (ie, not treating hypertension unless SBP >180 mm Hg) across several of these end points.

The SBP target after successful post-EVT reperfusion in patients with AIS-LVO has been a question of debate, with variability in management across centers.^6,7^ This debate stems from the controversy regarding the risks vs benefits of intensive SBP control in those patients. On one hand, it is hypothesized that more aggressive SBP control after successful reperfusion is associated with less risk of ICH.^8,23^ Moreover, higher SBP in the critical period after EVT could lead to cerebral hyperperfusion, resulting in neuroinflammation and cerebral edema.^12^ On the other hand, there may still be areas of focal hypoperfusion even after mechanical recanalization of the LVO with successful reperfusion. These focal areas of hypoperfusion could remain at risk of progressing to infarction with intensive BP reduction.^21^ Furthermore, patients who undergo EVT and achieve successful reperfusion may still have persistent venous postcapillary thrombosis. In such cases, aggressive reduction of SBP could potentially have an adverse impact on the prognosis of the acute ischemic lesion.^24^ The status of cerebral autoregulation in each patient may determine the risks and benefits of BP reduction after EVT, but this physiologic parameter cannot be practically and reliably measured in real time to guide BP management.

Observational data also offer arguments for both sides of the debate. Higher mean SBP during the first 24 hours after EVT is independently associated with higher odds of sICH, early neurologic deterioration, 3-month mortality, and worse 3-month functional outcomes.^11^ However, these findings cannot be considered proof that SBP reduction is beneficial. Meanwhile, greater BP variability after EVT has been associated with worse clinical outcomes,^25^ which would be an argument for exercising caution when lowering the BP in the acute postthrombectomy period.

With such heterogeneity in the literature about the risks vs benefits of intensive SBP control after EVT achieving successful reperfusion, a few RCTs were conducted to provide more robust evidence in this regard. In 2021, the results of the multicenter, open-label BP-TARGET (Blood Pressure Target in Acute Stroke to Reduce Hemorrhage After Endovascular Therapy) trial^21^ did not show significant differences between the intensive SBP target group (100-129 mm Hg) and the conventional care SBP target group (130-185 mm Hg) in terms of radiographic ICH (adjusted odds ratio [OR], 0.96 [95% CI, 0.60-1.51]; *P* = .84), hypotensive events, functional outcomes, and mortality. In 2022, the ENCHANTED2/MT (Second Enhanced Control of Hypertension and Thrombectomy Stroke Study),^22^ a multicenter open-label blinded–end point RCT, compared a more intensive SBP target (<120 mm Hg) with a less intensive SBP target (140-180 mm Hg), and the results still did not indicate any difference between the 2 groups in sICH or mortality. However, the more intensive SBP target had higher likelihoods of poor functional outcome (common OR, 1.37 [95% CI, 1.07-1.76]), early neurological deterioration (common OR, 1.53 [95% CI, 1.18-1.97]), and major disability at 90 days (OR, 2.07 [95% CI, 1.47-2.93]). In 2023, the results of both the BEST II (Blood Pressure After Endovascular Stroke Therapy-II)^12^ and OPTIMAL-BP (Outcome in Patients Treated With Intraarterial Thrombectomy–Optimal Blood Pressure Control)^13^ trials became available. The BEST II trial, a multicenter open-label blinded–end point futility RCT, compared the outcomes of 3 SBP targets after successful reperfusion: less than 140 mm Hg, less than 160 mm Hg, and less than 180 mm Hg. The study found that neither of the lower SBP targets (<140 and <160 mm Hg) met the predefined criteria for being ineffective or causing harm when considering the utility-weighted mRS score and the follow-up infarct volume.^12^ The OPTIMAL-BP trial, a multicenter open-label blinded–end point RCT, compared an intensive SBP target (<140 mm Hg) with a conventional SBP target (140-180 mm Hg) in patients with AIS-LVO and successful reperfusion, and a lower proportion of patients in the intensive SBP target group achieved functional independence (adjusted OR, 0.56 [95% CI, 0.33-0.96]; *P* = .03), with similar rates of mortality and sICH in both groups.^13^

### Limitations

This study has some limitations. The 4 RCTs included in our meta-analysis differed in multiple aspects, most notably the SBP target in their intensive arms. However, the conventional arms were similar (ie, SBP <180 mm Hg). Therefore, despite their differences, their combined results support a conservative strategy of using antihypertensive therapy only when the SBP exceeds 180 mm Hg.

## Conclusions

The findings of this systemic review and meta-analysis suggest that intensive SBP control does not result in better rates of functional independence, mortality, or sICH after EVT with successful reperfusion when compared with conventional SBP control (<180 mm Hg). Furthermore, the lack of any difference in the risk of sICH and trends toward higher risk of hypotensive events and lower probability of functional independence at 3 months argue against aggressive BP reduction after EVT. While awaiting the results of additional ongoing trials, a conservative BP management strategy after endovascular recanalization is recommended in daily practice.

## Notes

Supplement 1. **eTable 1.** Detailed Search Strategy **eTable 2.** Baseline Characteristics of Included Studies **eTable 3.** Blood Pressure Details of Included Studies **eFigure 1.** Risk of Bias Using the ROBINS-I Tool **eFigure 2.** Risk Percentage for Each Domain and Overall Risk of Bias Using the ROBINS-I Tool **eFigure 3.** Forest Plot of Baseline Characteristics for Dichotomous Variables Assessed **eFigure 4.** Forest Plot of Baseline Characteristics for Continuous Variables Assessed **eFigure 5.** Influence Analysis in Meta-Analysis Using Leave-One-Out Method

Supplement 2.
Data Sharing Statement
