# Error in Affiliations

**DOI:** 10.1001/jamanetworkopen.2024.49923

**Published:** 2024-11-15

In the Original Investigation titled “Intensive vs Conventional Blood Pressure Control After Thrombectomy in Acute Ischemic Stroke: A Systematic Review and Meta-Analysis,”^1^ published February 22, 2024, the affiliation listed for Dr Mortezaei was incorrectly listed as Department of Radiology, Mayo Clinic, Rochester, Minnesota; the correct affiliation is Gonabad University of Medical Sciences, Gonabad, Iran. This article has been corrected.
