# Supplementary material for: Intensive vs Conventional Blood Pressure Control After Thrombectomy in Acute Ischemic Stroke: A Systematic Review and Meta-Analysis
Source: JAMA Netw Open. 2024 Feb 22;7(2):e240179. doi: 10.1001/jamanetworkopen.2024.0179 (PMC10884884; doi:10.1001/jamanetworkopen.2024.0179)
Supplement: Supplement 1. — eTable 1. Detailed Search Strategy eTable 2. Baseline Characteristics of Included Studies eTable 3. Blood Pressure Details of Included Studies eFigure 1. Risk of Bias Using the ROBINS-I Tool eFigure 2. Risk Percentage for Each Domain and Overall Risk of Bias Using the ROBINS-I Tool eFigure 3. Forest Plot of Baseline Characteristics for Dichotomous Variables Assessed eFigure 4. Forest Plot of Baseline Characteristics for Continuous Variables Assessed eFigure 5. Influence Analysis in Meta-Analysis Using Leave-One-Out Method [file jamanetwopen-e240179-s001.pdf]

Supplementary Online Content

Ghozy S, Mortezaei A, Elfil M, et al. Intensive vs conventional blood pressure control after thrombectomy in acute ischemic stroke: a systematic review and meta-analysis. *JAMA Netw Open*. 2024;7(2):e240179. doi:10.1001/jamanetworkopen.2024.0179

eTable 1. Detailed Search Strategy

eTable 2. Baseline Characteristics of Included Studies

eTable 3. Blood Pressure Details of Included Studies

eFigure 1. Risk of Bias Using the RoB 2 Tool

eFigure 2. Risk Percentage for Each Domain and Overall Risk of Bias Using the RoB 2 Tool

eFigure 3. Forest Plot of Baseline Characteristics for Dichotomous Variables Assessed

eFigure 4. Forest Plot of Baseline Characteristics for Continuous Variables Assessed

eFigure 5. Influence Analysis in Meta-Analysis Using Leave-One-Out Method

This supplementary material has been provided by the authors to give readers additional information about their work.

© 2024 Ghozy S et al. *JAMA Network Open*.

eTable 1. Detailed Search Strategy

Pubmed
(blood pressure[Title/Abstract]) AND ("endovascular therapy"[Title/Abstract] OR "endovascular thrombectomy"[Title/Abstract] OR "Thrombectomy"[Title/Abstract]) AND ("Stroke"[Title/Abstract]) AND "Trial"[Title/Abstract]
Date: September 8th
Results: 69

Scopus
(TITLE-ABS("blood pressure")) AND (TITLE-ABS("endovascular therapy") OR TITLE-ABS("endovascular thrombectomy") OR TITLE-ABS(Thrombectomy)) AND (TITLE-ABS(Stroke)) AND TITLE-ABS(Trial)
Date: September 8th
Results: 118

Embase <1974 to 2023 September 8>
("blood pressure".tw.) AND ("endovascular therapy".tw. OR "endovascular thrombectomy".tw. OR Thrombectomy.tw.) AND (Stroke.tw.) AND Trial.tw.
Date: September 8th
Results: 102

# Web of Science Search Strategy (v0.1)
Database: All Databases
Entitlements:
- WOS: 1900 to 2023
- BCI: 1969 to 2023
- CCC: 1998 to 2023
- DRCI: 1993 to 2023
- DIIDW: 1993 to 2023
- KJD: 1980 to 2023
- MEDLINE: 1950 to 2023
- PPRN: 1991 to 2023
- PQDT: 1637 to 2023
- SCIELO: 2002 to 2023
- ZOOREC: 1993 to 2023

Searches:
1: ((TI="blood pressure" OR AB="blood pressure")) AND ((TI="endovascular therapy" OR AB="endovascular therapy") OR (TI="endovascular thrombectomy" OR AB="endovascular thrombectomy") OR (TI=Thrombectomy OR AB=Thrombectomy)) AND ((TI=Stroke OR AB=Stroke)) AND (TI=Trial OR AB=Trial) and Preprint Citation Index (Exclude – Database)

Date: September 8th
Results: 115

© 2024 Ghozy S et al. *JAMA Network Open*.

Cochrane Library
("blood pressure":ti,ab) AND ("endovascular therapy":ti,ab OR "endovascular thrombectomy":ti,ab OR Thrombectomy:ti,ab) AND (Stroke:ti,ab) AND Trial:ti,ab
Date: September 8th
Results: 95

© 2024 Ghozy S et al. *JAMA Network Open*.

eTable 2. Baseline Characteristics of Included Studies

Study		Mazighi et al., 2021		Mistry et al.,2023			Nam et al., 2023		Yang et al., 2022	
Design		RCT		RCT			RCT		RCT	
Groups		Intensive SBP target (100–129 mm Hg)	Standard SBP target (130–185 mm Hg)	Intensive SBP target (<140mmHg)	Intensive SBP target (<160mmHg)	Standard SBP target (≤180mmHg)	Intensive SBP target (<140mmHg)	Standard SBP target (140-180mmHg)	Intensive SBP target (<120 mm Hg)	Standard SBP target (140-180mmHg)
No of Patients		158	160	40	40	40	155	147	407	409
Male/female		81/77	72/88	28-Dec	19/23	20/20	92/63	88/59	249/158	257/152
Age	Mean	76	73.3	75.2	69.4	67.4	73.2	72.9	68	67
	SD	14.2	14.96	15.8	10.9	14	12.1	10.8	12	12
Past Medical History	Hypertension	110	113	32	28	32	121	110	267	261
	DM	34	33	12	15	13	65	62	81	82
	Smoking	19	24	8	12	10	39	29	69	73
	Hypercholesterolaemia	59	55	33	28	34	61	54	14	13
	Previous Stroke/TIA	25	21	N/A	N/A	N/A	36	30	107	139
	Antiplatelets	44	37	14	13	19	N/A	N/A	34	39
	Anticoagulant	36	34	10	3	9	N/A	N/A	20	20
	Atrial Fibrillation	N/A	N/A	19	13	21	77	69	84	98
	Cronoray Artery Obstructive disease	N/A	N/A	N/A	N/A	N/A	18	16	51	59
	Active Cancer	N/A	N/A	N/A	N/A	N/A	9	5	N/A	N/A
Race and ethnicity	Cradiac heart failure	N/A	N/A	N/A	N/A	N/A	7	7	N/A	N/A
	Black	N/A	N/A	2	2	5	N/A	N/A	N/A	N/A
	Hispanic or Latino	N/A	N/A	0	0	2	N/A	N/A	N/A	N/A
	Native Hawaiian or Other Pacific Islander	N/A	N/A	1	0	0	N/A	N/A	N/A	N/A
	Not Hispanic or Latino ethnicity	N/A	N/A	39	39	36	N/A	N/A	N/A	N/A
	White	N/A	N/A	34	37	34	N/A	N/A	N/A	N/A
	Multiracial	N/A	N/A	3	0	0	N/A	N/A	N/A	N/A

© 2024 Ghozy S et al. *JAMA Network Open*.

	Chinese	N/A	N/A	N/A	N/A	N/A	N/A	N/A	407	409
Antihypertensive drugs at admission	CCB	40	27	25	17	7	107	28	72	61
	B blocker	46	58	4	4	2	10	0	12	16
	Diuretics	40	40	N/A	N/A	N/A	N/A	N/A	15	10
	ACEi	31	33	N/A	N/A	N/A	N/A	N/A	41	47
	ARB	34	27	N/A	N/A	N/A	N/A	N/A	N/A	N/A
	Vasodilators	7	2	0	1	1	N/A	N/A	N/A	N/A
	Central agonist	5	2	Comment	N/A	N/A	N/A	N/A	N/A	N/A
Occlusion site	MCA	117	119	37	33	38	108	101	197	209
	ACA	N/A	N/A	N/A	N/A	N/A	1	3	8	7
	BA	N/A	N/A	N/A	N/A	N/A	12	12	47	38
	VA	N/A	N/A	N/A	N/A	N/A	4	3	7	7
	PCA	N/A	N/A	N/A	N/A	N/A	0	1	7	4
	TICA	N/A	N/A	N/A	N/A	N/A	N/A	N/A	30	29
	ICA	41	39	7	9	5	34	32	29	24
Cause of large-vessel occlusion	Large vessel atherosclerosis	N/A	N/A	N/A	N/A	N/A	41	43	197	224
	Cardioembolism	N/A	N/A	N/A	N/A	N/A	76	76	145	137
	Dissection	N/A	N/A	N/A	N/A	N/A	N/A	N/A	7	6
	Uncertain	N/A	N/A	N/A	N/A	N/A	34	27	58	41
General Anesthesia		2	7	8	8	7	N/A	N/A	173	172
No of IV thrombolysis		85	83	17	19	19	44	54	132	115
NIHSS	Mean	16.7	16.7	16.7	18.7	14	11.32	9.01	15	15
	SD	5.98	5.2	4.2	6.15	4.6	7.2	6.99	7.43	7.43
ASPECTS score	Mean	N/A	N/A	7.7	7	8	N/A	N/A	N/A	N/A
	SD	N/A	N/A	2.3	1.53	1.53	N/A	N/A	N/A	N/A
Adverse Events	Recurrent ischemic stroke	0	1	N/A	N/A	N/A	N/A	N/A	25	20
	stroke worsening	1	1	N/A	N/A	N/A	N/A	N/A	N/A	N/A
	Hemicraniotomy	1	1	N/A	N/A	N/A	N/A	N/A	14	15

© 2024 Ghozy S et al. *JAMA Network Open*.

	Neurological-related Mortality	8	7	3	6	3	N/A	N	N/A	N/A
	Non-neurological-related mortality Mortality	3	0	N/A	N/A	N/A	N/A	N	N/A	N/A
	Mortality at 3months	29	21	7	14	6	12	7	66	61
Hemorrhagic transformation	None	N/A	N/A	N/A	N	N/A	77	77	N/A	N/A
	HI1	N/A	N/A	N/A	N/A	N/A	27	24	N/A	N/A
	HI2	N/A	N/A	N/A	N/A	N/A	17	19	N/A	N/A
	PH1	N/A	N/A	N/A	N/A	N/A	17	13	N/A	N/A
	PH2	N/A	N/A	N/A	N/A	N/A	17	17	N/A	N/A
Infarction volumes(mL)	Mean	N/A	N/A	32.4	50.7	46.4	60.8	42.1	111	106
	SD	N/A	N/A	N/A	N/A	N/A	91.6	73.9	86.54	92.5

© 2024 Ghozy S et al. *JAMA Network Open*.

eTable 3. Blood Pressure Details of Included Studies

Study		Mizaghi et al., 2021		Mistry et al., 2023			Nam et al., 2023		Yang et al., 2022	
Groups		Intensive SBP target (100–129 mm Hg)	Standard SBP target (130–185 mm Hg)	Intensive SBP target (<140mmHg)	Intensive SBP target (<160mmHg)	Standard SBP target (≤180mmHg)	Intensive SBP target <140mmHg	Standard SBP target (140-180mmHg)	Intensive SBP target (<120 mm Hg)	Standard SBP target (140-180mmHg)
SBP 24H	Mean	128	138	122	130	129	129.2	138	120.8	139
	SD	11	17	15	18	20	7.7	13.6	12.99	17.92
Min SPB 24H		98	109	N/A	N/A	N/A	N/A	N/A	N/A	N/A
Max SBP 24H		166	170	N/A	N/A	N/A	N/A	N/A	N/A	N/A
DBP 24H	Mean	N/A	N/A	66	74	75	72	77	66.9	78.4
	SD	N/A	N/A	12	15	16	8.1	9.9	11.96	14.38
No of Measurement taken	Mean	15.9	14.8	N/A	N/A	N/A	N/A	N/A	N/A	N/A
	SD	5.1	5.8	N/A	N/A	N/A	N/A	N/A	N/A	N/A
Proportion of time spent below target	<140mmHg	96 (61%)	48 (30%)	34 (85%)	N/A	N/A	128 (83%)	81(54.2%)	N/A	N/A
	140-180mmHg	46 (29.5%)	105 (66.6%)	N/A	37(92%)	N/A	22 (14.2%)	63(42.1%)	N/A	N/A
	<180mmHg	N/A	N/A	N/A	N/A	39(99%)	154 (99.6%)	148 (99.1%)	N/A	N/A

© 2024 Ghozy S et al. *JAMA Network Open*.

eFigure 1. Risk of Bias Using the RoB 2 Tool

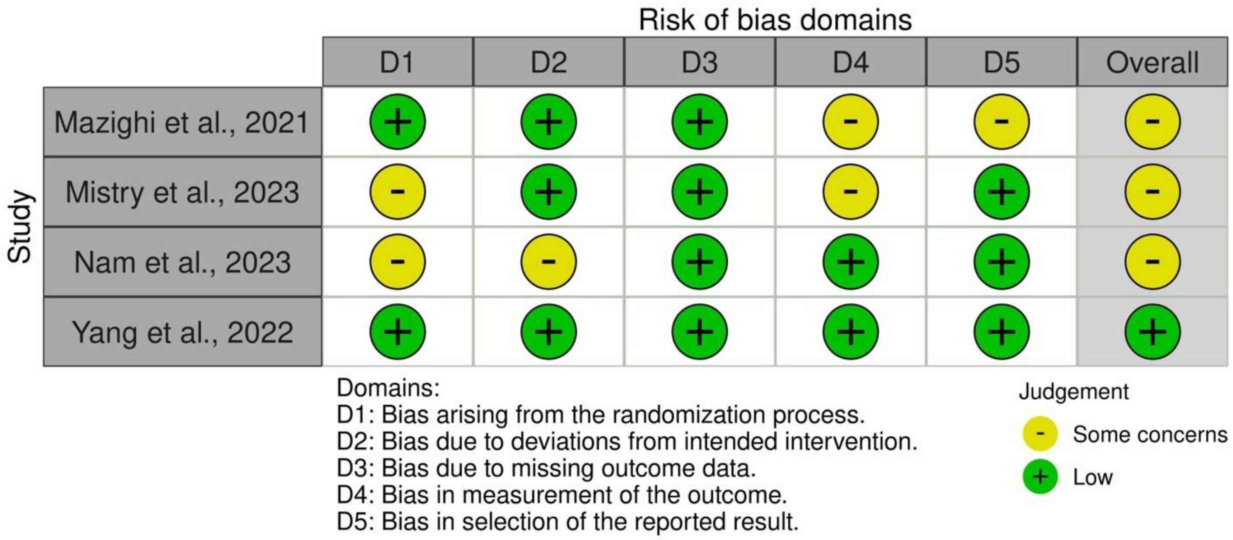

© 2024 Ghozy S et al. *JAMA Network Open*.

eFigure 2. Risk Percentage for Each Domain and Overall Risk of Bias Using the RoB 2 Tool

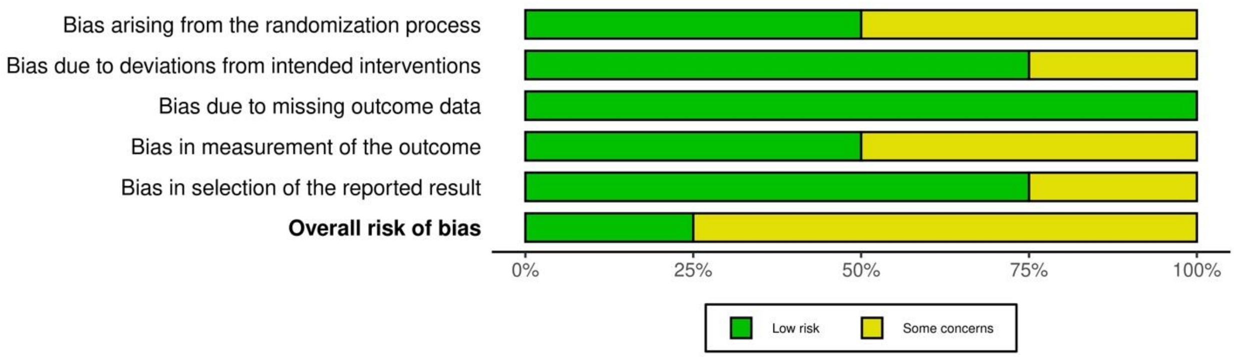

© 2024 Ghozy S et al. *JAMA Network Open*.

eFigure 3. Forest Plot of Baseline Characteristics for Dichotomous Variables Assessed

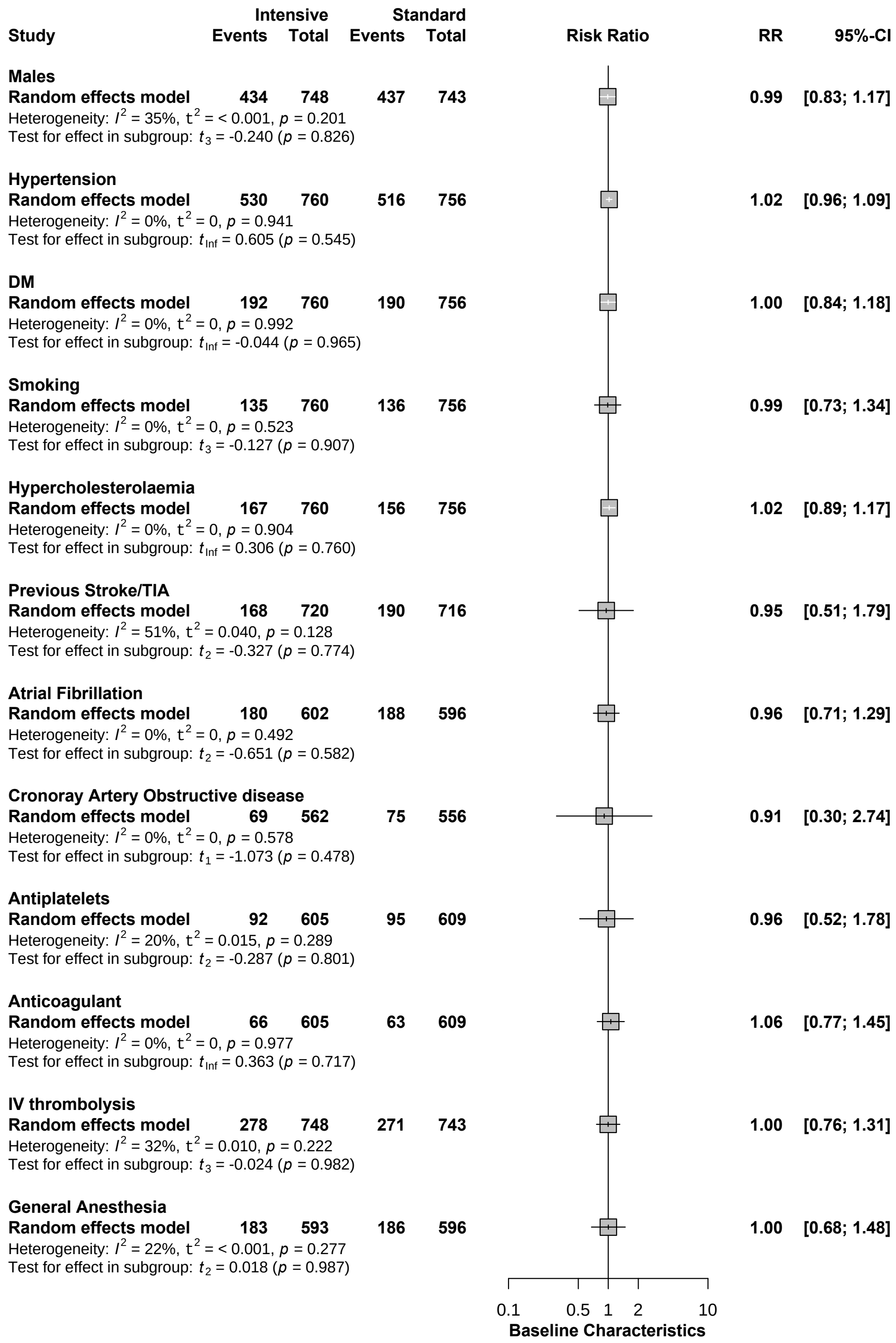

© 2024 Ghozy S et al. *JAMA Network Open*.

eFigure 4. Forest Plot of Baseline Characteristics for Continuous Variables Assessed

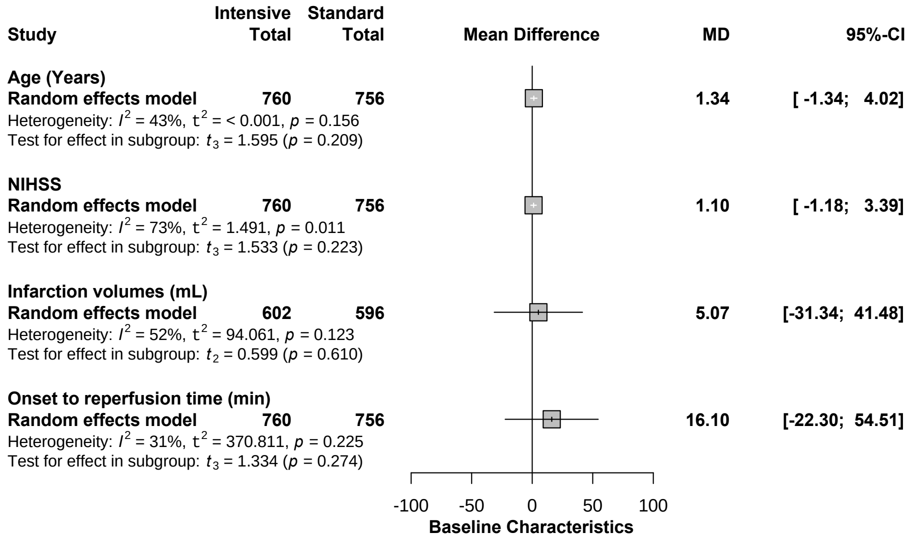

© 2024 Ghozy S et al. *JAMA Network Open*.

eFigure 5. Influence Analysis in Meta-Analysis Using Leave-One-Out Method

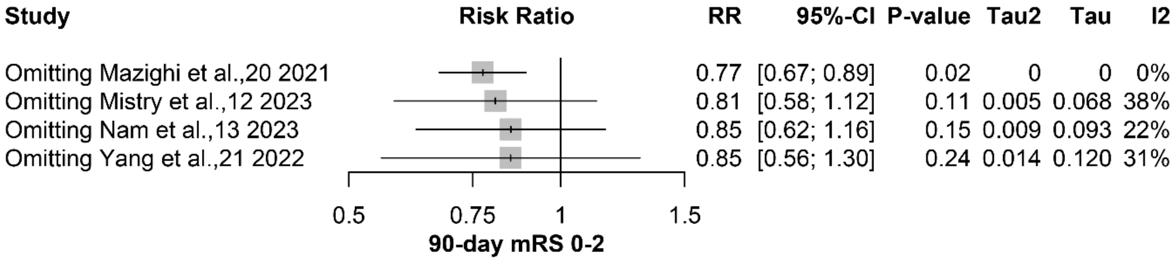

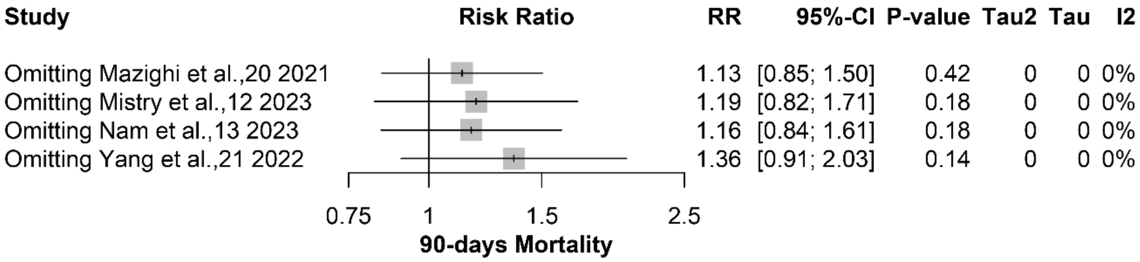

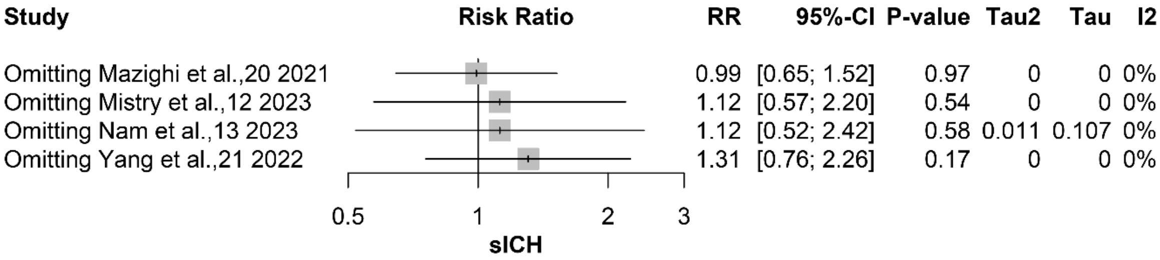

© 2024 Ghozy S et al. *JAMA Network Open*.
