# Supplementary material for: Intensive vs Conventional Blood Pressure Control After Thrombectomy in Acute Ischemic Stroke: A Systematic Review and Meta-Analysis
Source: JAMA Netw Open. 2024 Feb 22;7(2):e240179. doi: 10.1001/jamanetworkopen.2024.0179 (PMC10884884; doi:10.1001/jamanetworkopen.2024.0179)
Supplement: Supplement 2. — Data Sharing Statement [file jamanetwopen-e240179-s002.pdf]

Data Sharing Statement

Ghozy. Intensive vs Conventional Blood Pressure Control After Thrombectomy in Acute Ischemic Stroke. *JAMA Netw Open*. Published February 22, 2024.
doi:10.1001/jamanetworkopen.2024.0179

Data
Data available: No
